# Advances in Research on Brain Structure and Activation Characteristics in Patients with Anterior Cruciate Ligament Reconstruction: A Systematic Review

**DOI:** 10.3390/brainsci15080831

**Published:** 2025-08-01

**Authors:** Jingyi Wang, Yaxiang Jia, Qiner Li, Longhui Li, Qiuyu Dong, Quan Fu

**Affiliations:** School of Kinesiology and Health, Capital University of Physical Education and Sports, Beijing 100191, China; wangjingyi2023@cupes.edu.cn (J.W.); lqe0613@163.com (Q.L.);

**Keywords:** anterior cruciate ligament reconstruction, brain, cortex activation, neuroplasticity

## Abstract

**Objectives**: To synthesize evidence on structural and functional neuroplasticity in patients after anterior cruciate ligament reconstruction (ACLR) and its clinical implications. **Methods:** Adhering to the PRISMA guidelines for systematic reviews and meta-analyses, a literature search was conducted using PubMed, Embase, Web of Science, Scopus, and Cochrane CENTRAL (2018–2025) using specific keyword combinations, screening the results based on predetermined inclusion and exclusion criteria. **Results**: Among the 27 included studies were the following: (1) sensory cortex reorganization with compensatory visual dependence (5 EEG/fMRI studies); (2) reduced motor cortex efficiency evidenced by elevated AMT (TMS, 8 studies) and decreased γ-CMC (EEG, 3 studies); (3) progressive corticospinal tract degeneration (increased radial diffusivity correlating with postoperative duration); (4) enhanced sensory-visual integration correlated with functional recovery. **Conclusions:** This review provides a novel synthesis of evidence from transcranial magnetic stimulation (TMS), electroencephalography (EEG), functional near-infrared spectroscopy (fNIRS), diffusion tensor imaging (DTI), and functional magnetic resonance imaging (fMRI) studies. It delineates characteristic patterns of post-ACLR structural and functional neural reorganization. Targeting visual–cognitive integration and corticospinal facilitation may optimize rehabilitation.

## 1. Introduction

Anterior cruciate ligament (ACL) injuries are among the most severe lower extremity sports injuries [1]. As sports participation increases, the incidence of ACL tears is rising, representing approximately 56% of all knee ligament injuries [1]. The ACL, rich in mechanoreceptors [2], is crucial for proprioceptive feedback and maintaining knee joint function. ACL rupture results in static knee joint instability due to anatomical structure destruction and dynamic functional imbalance caused by proprioceptive abnormalities [3]. Despite ACLR restoring structural knee joint stability, patients frequently suffer from persistent dynamic functional imbalance postoperatively, severely impacting motor function recovery [4]. Consequently, rehabilitation programs must integrate exercise training and educational interventions, alongside conservative treatment or ACLR, to optimize function and facilitate return to sports [5]. While conservative treatment and surgery demonstrate significant mid-term efficacy in young, active populations [6], the re-injury rate remains high (29.5%), predominantly within two years of sports resumption [7]. The high risk of re-injury is not confined to the reconstructed limb but also significantly impacts the contralateral healthy knee joint. Research indicates that the graft ligament tear rate post-ACLR is 5.86% (at 2.64 years), while the contralateral ACL tear rate is 6.66% (at 2.78 years) [8]. Furthermore, within 5–10 years, approximately 50% of patients may develop early osteoarthritis in the tibiofemoral and patellofemoral joints [9,10]. Recent studies confirm significant differences in bilateral lower extremity motor control between ACL-injured/reconstructed patients and healthy individuals [11,12,13,14]. Abnormal motor control post-ACLR may further elevate the risk of joint degeneration. Research has confirmed that ACLR patients exhibit an elevated peak knee abduction moment during the gait cycle, a biomechanical change strongly linked to osteoarthritis (OA) progression [15]. Additionally, quadriceps functional inhibition resulting from joint-derived muscle inhibition post-ACL injury may promote post-traumatic OA development by diminishing gait energy absorption capacity [16]. These biomechanical and neuromuscular abnormalities can result in abnormal cartilage load distribution, thereby initiating joint degeneration [17].

Recent neuroscience research suggests that, while ACL injury does not directly harm the brain’s nervous system, it impairs ligament mechanoreceptors, disrupting afferent signals [18] and compensatory motor responses [19], thereby altering supraspinal activity and excitability. Within the sensory system, information from various sources can compensate for one another [20]. When specific inputs change, the central nervous system may depend on more reliable information sources to maintain postural control. Furthermore, when proprioceptive input changes, the central nervous system may enhance sensory integration and activate higher-level cortical functions to plan and control movements, compensating for proprioceptive deficits. Recent research indicates that the ACL demonstrates significant anatomical structural heterogeneity in its healing potential, with its biological regenerative capacity exhibiting a longitudinal gradient. According to the Epiligament (EL) theory, the proximal ACL region exhibits superior regenerative capacity relative to the distal region, attributed to its ligament sheath structure, which is rich in neurovascular bundles. This disparity arises from a marked reduction in neurovascular distribution within the distal region [21,22]. Following reconstruction, this regional disparity, combined with variations in graft types, results in uneven reinnervation of the distal grafts. This, in turn, induces persistent proprioceptive deficits, thereby exacerbating central compensatory adaptations [23]. Significant changes in the central nervous system of ACLR patients can result in alterations to neural pathways and movement patterns of the contralateral limb [24]. Changes in afferent information due to the injury can lead to alterations in neural activity, potentially resulting in structural changes in the central nervous system over time. Persistent changes in cortical tissue morphology or function are termed neuroplasticity. These changes may persist long after ACLR, impairing knee function and heightening the risk of bilateral injuries. However, although these neural changes can sustain basic motor functions under static conditions, during movement, the high cognitive demands triggered by external disturbances or unpredictable environments may rapidly surpass the capacity for complex motor coordination, resulting in neuromuscular control failure [25]. This likely contributes significantly to the heightened risk of graft rupture and contralateral reinjury following ACLR. A deeper understanding of the unique neural activity characteristics in post-ACLR patients can elucidate the causes of their clinical symptoms and functional impairments. This knowledge may also identify new targets for rehabilitation therapy, thereby more effectively enhancing postoperative knee function in ACLR patients and mitigating the risk of secondary injuries and complications. Given that previous review papers were published over five years ago, an updated review is imperative. This systematic review synthesizes the research findings from 2018 to 2025 across three core domains:

(1) Typical patterns of structural brain remodeling and alterations in functional brain activation following ACLR;
(2) The relationship between neuroadaptive changes and subsequent clinical functional outcomes;
(3) Methodological limitations within the field of neurorehabilitation and their implications for translational applications.

By examining these core domains, this review seeks to elucidate the neurophysiological basis underlying persistent functional deficits after ACLR and to identify potential targets for neuromodulation interventions.

## 2. Methods

### 2.1. Study Design

The protocol for this systematic review was prospectively registered in the International Prospective Register of Systematic Reviews (registration number: CRD420251109648). This study performed a systematic review of experimental research in accordance with the PRISMA guidelines and utilized the Population, Intervention, Comparison, Outcome (PICO) framework to formulate the research question (see Supplementary Table S1 for details) [26]. Population: Individuals who had undergone ACL reconstruction (ACLR); Intervention: Utilization of neuroimaging techniques as an assessment tool; Comparison: Comparison with age- and gender-matched control groups or between involved and uninvolved limbs; Outcome: Evaluation of brain structure and functional performance indicators.

### 2.2. Search Methods

A systematic literature search was performed in PubMed, Embase, Web of Science, Scopus, and Cochrane CENTRAL covering the period from 1 January 2018 to 31 May 2025, restricted to English-language publications. The search utilized various combinations of the following terms: [(“Anterior cruciate ligament” OR “ACL” OR “Anterior cruciate ligament reconstruction”) AND (“brain” OR “cortex” OR “central nervous system” OR “nervous system”) AND (“change” OR “plasticity” OR “neuroplasticity” OR “modifications”)].

### 2.3. Eligibility Criteria

The inclusion and exclusion criteria were jointly established by two authors and independently reviewed and assessed. Studies were selected based on the following inclusion criteria: (1) Language: Articles written in English only; (2) Publication type: Research or journal articles; (3) Publication status: Published articles with full text available; (4) Participants: Patients with anterior cruciate ligament reconstruction; (5) Outcome measures: Studies with indicators evaluating brain structure and function; (6) Study type: Controlled experimental studies. The search period for publications was from January 2018 to 31 May 2025.

### 2.4. Selection Process

All of the retrieved literature was managed using EndNote 20. Following automated duplicate removal via EndNote’s algorithm, two researchers (J.W. and Q.L.) performed independent blinded screening of titles/abstracts using custom-defined inclusion/exclusion tags in EndNote. Full texts of potentially eligible studies were retrieved and similarly screened. Disagreements were resolved through consensus discussion with a third researcher (Y.J.), with documented audit trails maintained in EndNote.

### 2.5. Extraction of Data

The two authors independently extracted the following information from the included studies: (1) study details, including author names and publication year; (2) participant characteristics, including age, gender, injury/postoperative duration, and type of injury/surgery; (3) technical specifications; (4) test tasks; (5) main research findings.

### 2.6. Risk of Bias Assessment

To evaluate the methodological quality of the included studies, we employed the Risk Of Bias In Non-randomized Studies of Interventions (ROBINS-I) tool [27]. This tool assesses bias across seven domains: (1) confounding bias; (2) selection bias; (3) classification of interventions bias; (4) deviations from intended interventions; (5) missing data bias; (6) measurement of outcomes bias; (7) selective reporting bias.

Two independent reviewers (J.W. and Q.L.) conducted assessments, with disagreements resolved via consensus or third reviewer (Y.J.) consultation. Domains were rated Low, Moderate, High, or Unclear risk. Overall risk was classified as follows: (1) Low: All domains low risk; (2) Moderate: ≥1 domain moderate risk; (3) High: ≥1 domain high risk or multiple moderate risks. Results informed narrative synthesis (the figure in Section 3.4).

### 2.7. Statistical Analysis

Given significant heterogeneity across studies, meta-analysis was precluded. We adhered to the PRISMA guidelines for systematic reviews without meta-analysis (SWiM) [28]. Extracted data were tabulated (Table 1) using Microsoft Excel. Continuous variables (e.g., age, postoperative duration) are reported as mean ± standard deviation (SD). Correlational findings from primary studies were synthesized narratively. Patterns of neural alterations (Table 2) were quantified by tallying recurrent findings across studies.

## 3. Results

### 3.1. Study Selection

**Figure 1:**
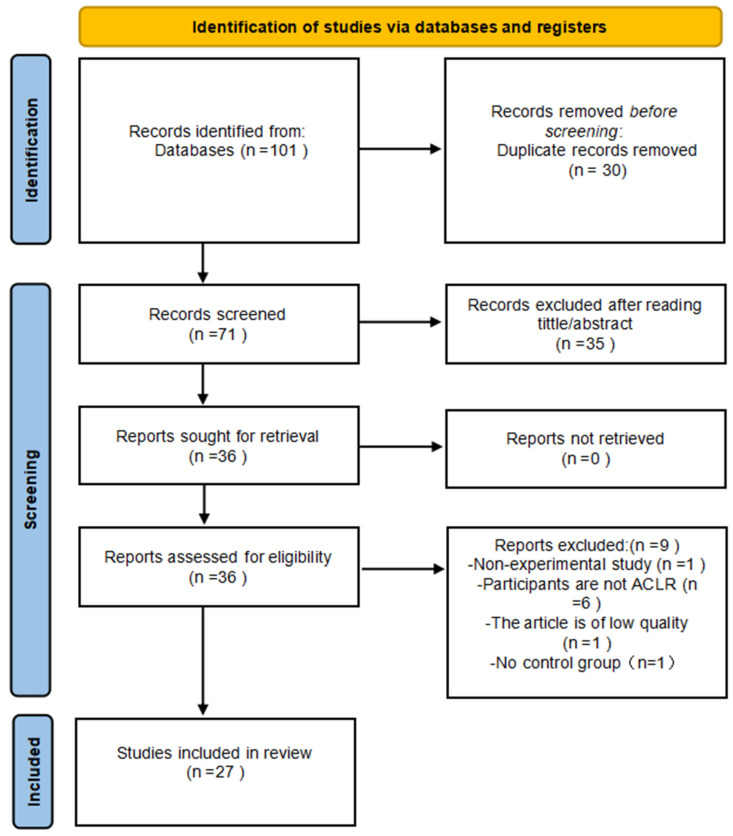
Flow chart illustrating the following steps of the study selection.

A total of 101 articles were retrieved from English-language electronic databases. In the initial phase, duplicates were removed, resulting in 71 studies screened based on titles and abstracts. Of these, 35 studies met the criteria for further evaluation. The full texts were then retrieved and assessed. Using predefined inclusion and exclusion criteria, the 35 studies were independently evaluated, resulting in the exclusion of 9 studies. The remaining 27 studies were deemed eligible for inclusion in this review. The authors followed all steps of the PRISMA flowchart and selected these studies as the sample for further discussion to support this research. Based on these 27 studies, the authors constructed this systematic literature review for academic reference and further research. Figure 1 illustrates the search and screening process (see Supplementary Figure S1 for details).

### 3.2. Characteristics of Included Studies

A total of 27 studies were included in this systematic review.

Of the 27 included studies, 2 [43,49] solely compared differences between the injured and non-injured sides. The remaining 25 studies incorporated a healthy control group, including one longitudinal study that assessed differences across various time points and compared them with healthy individuals [37].

Concerning participant gender, one study [32] enrolled exclusively male participants, while two studies [46,47] recruited solely female participants. One study [29] did not disclose gender information. The rest of the studies included both genders. The average age of participants predominantly ranged from 20 to 30 years, with only two studies [41,56] including participants over 30. All studies encompassed younger age cohorts.

Concerning the postoperative timing of participant selection, four studies enrolled patients in the early postoperative phase (within 3 months). Notably, one study [37] assessed changes in multiple indicators between patients within 3 months and those beyond 3 months postoperatively. Three studies [39,40,55] selected patients approximately six months postoperatively. The majority of studies included patients in the late postoperative phase.

Concerning surgical techniques and graft selection, eight studies [29,31,43,47,49,51,52,53,55] omitted specific details, two studies [30,48] utilized hamstring grafts, while the remaining studies employed a variety of graft types.

Concerning the surgical limb side, 12 studies [29,31,32,33,34,35,36,38,39,40,43,52] omitted details on the injured side. One study [37] characterized the ACLR side as either dominant or non-dominant. Six studies enrolled participants with exclusively left-sided [41,44,45,47,51] or right-sided [55] ACLR, while the remaining studies included participants with both left and right-sided ACLR.

The neuroimaging techniques utilized in the included studies encompass electroencephalography (EEG), transcranial magnetic stimulation (TMS), magnetic resonance imaging (MRI), and functional near-infrared spectroscopy (fNIRS).

Eleven studies utilized EEG to evaluate brain function. Specifically, Sherman et al. [33] investigated the lateralized readiness potential (LRP) during a seated lateralized choice reaction time task. Sherman et al. [38], Riehm et al. [36], and Sherman et al. [34] analyzed the coherence between EEG and EMG signals within specific frequency bands during force control. Furthermore, seven studies performed EEG spectral analysis, encompassing test tasks such as joint angle reproduction, joint force reproduction, single-leg standing, passive joint movement, and passing tasks.

Three studies [40,42,43] employed TMS to evaluate the active motor threshold (AMT), thereby assessing corticospinal excitability.

Fourteen studies utilized MRI, with two employing diffusion tensor imaging (DTI) to assess the corticospinal tract volume and one utilizing diffusion-weighted imaging (DWI) to analyze white matter microstructure. Eleven studies employed functional magnetic resonance imaging (fMRI), encompassing resting-state scans [41], periodic knee and hip extension/flexion movements akin to heel slides [44], picture observation and motor imagery tasks [47,51], and knee joint position sense (JPS) assessments [48]. The remaining six studies conducted independent periodic knee flexion and extension movements ranging from 0° to 45°.

One study [55] employed fNIRS to monitor alterations in cerebral blood flow among ACLR patients during repetitive stair-climbing tasks.

### 3.3. Synthesis of Findings

Despite initial plans to conduct a meta-analysis, significant heterogeneity among the included studies precluded pooling of data. The primary sources of heterogeneity were as follows:

(1) Methodological diversity: Varied neuroimaging techniques (e.g., EEG, TMS, fMRI, fNIRS) measured fundamentally distinct neural phenomena;
(2) Outcome metric inconsistency: Studies reported non-comparable units (e.g., % signal change vs. threshold values);
(3) Clinical heterogeneity: Substantial variations in patient characteristics (38% unreported graft types; postoperative duration range: 1.5 months to 5.5 years) and control group designs (healthy controls vs. within-subject uninjured limbs).

Consequently, a narrative synthesis approach was adopted in accordance with PRISMA guidelines for systematic reviews without meta-analysis (SWiM).

Quantitative analysis of the 27 included studies revealed a consistent pattern of neurological changes (see Table 2 for details). Impaired motor cortical efficiency was the most frequently reported finding, documented in 8 of the 27 studies. This was characterized by elevated active motor thresholds as measured by TMS, abnormal EEG spectral power, and reduced fMRI activation. Five studies confirmed the presence of visual dependency. Three studies observed structural degeneration, including corticospinal tract atrophy and cortical thinning. Notably, Yu et al. [56] reported progressive white matter deterioration, indicated by increased RD values, which correlated positively with postoperative duration (r = 0.623, *p* < 0.001). Clinically, two studies found that enhanced sensory/visual integration, as observed via fMRI, was associated with improved International Knee Documentation Committee (IKDC) scores and reduced proprioceptive errors.

### 3.4. Risk of Bias Assessment of the Included Literature

**Figure 2:**
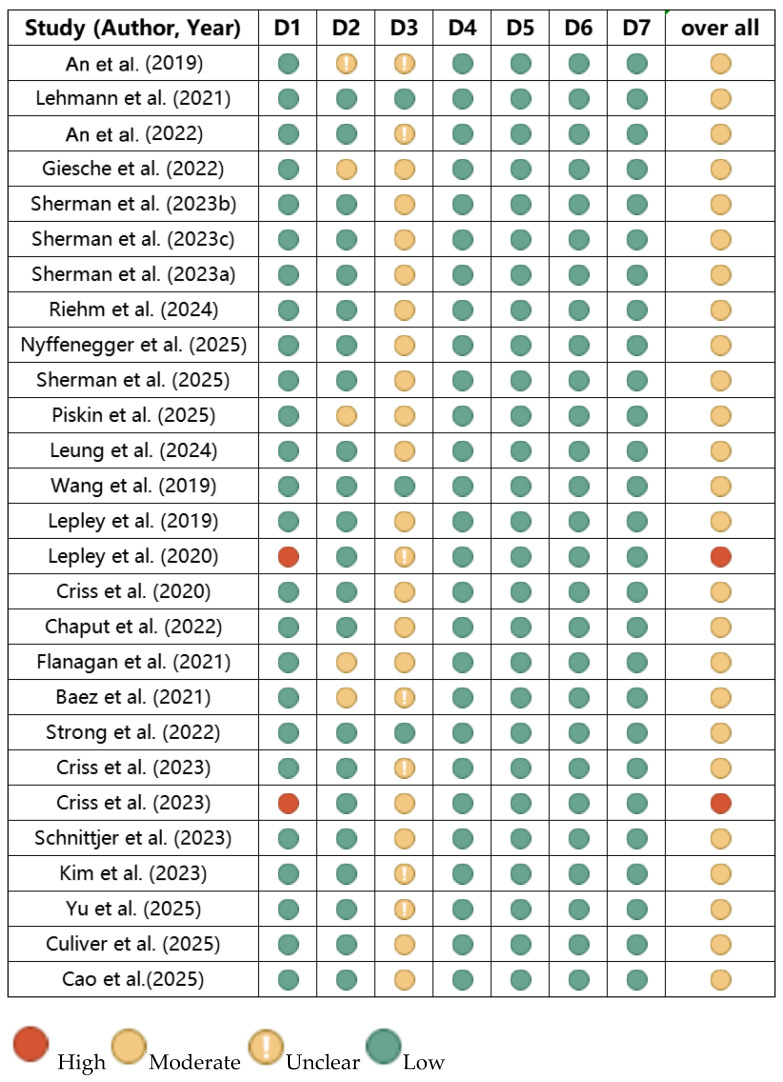
Risk of bias summary [29,30,31,32,33,34,35,36,37,38,39,40,41,42,43,44,45,46,47,48,49,50,51,52,53,54,55]. D1: Risk of bias due to confounding; D2: Risk of bias in selection; D3: Risk of bias in intervention class; D4: Risk of bias due to deviations from intended interventions; D5: Risk of bias due to missing data; D6: Risk of bias arising from measurement of the outcome; D7: Risk of bias in selection of the reported result.

Across the 27 included studies, the assessment revealed varying levels of bias risk across different domains. Regarding confounding bias, the majority of studies adequately controlled for key confounders such as gender, age, and activity level. For selection bias, two self-controlled studies (comparing affected vs. healthy sides) were judged to be at high risk, while moderate risk was identified in four studies due to selective sampling (one recruiting only athletes, two only females, and one only males); additionally, the risk was unclear in one study that did not report participant gender. In terms of intervention classification bias related to graft type, there were three studies specified that a single graft type was at low risk, sixteen studies that did not control for graft type were at moderate risk, and the risk was unclear in eight studies that did not report the graft type used. For all other domains assessed—deviations from intended interventions, missing data, outcome measurement, and selective reporting—all studies were judged to be at low risk. The overall distribution of bias risk across the studies is summarized in Figure 2.

## 4. Discussion

### 4.1. Characteristics of Cortical Activity Changes in ACLR

#### 4.1.1. Altered Somatosensory Motor Cortex Activity

The sensory center comprises the primary sensory area (S1), situated in the postcentral gyrus and the posterior segment of the parietal lobe, along with the secondary sensory area (SII) located beneath it, extending into the upper region of the parietal lobe. An et al. [29] investigated the brain activity of ACLR patients during a three-second anterior tibial translation task. The findings indicated that, compared to the control group and healthy limbs, the sensory cortical activity of the ACLR limb increased during early joint loading, yet no significant differences were observed among groups during early joint relaxation. During late joint loading, the sensory cortical activity of the ACLR limb exceeded that of the control group, although no significant differences were noted between limbs. The elevated desynchronized α-2 activity in the sensory cortex signifies enhanced sensory processing in ACLR patients, potentially improving proprioceptive awareness, assisting in motor planning adjustments, and facilitating muscle contraction, thereby better maintaining joint stability. Despite similar knee laxity in ACLR patients compared to healthy individuals, their cortical activation patterns diverge from those of healthy individuals, compensating for alterations in peripheral sensory input resulting from damage to joint mechanoreceptors. An et al. [31] observed a decrease in alpha-2 power in the central somatosensory cortex of ACLR patients during single-leg standing, implying that ACLR patients require enhanced sensory input from the lower limb joints.

Functional magnetic resonance imaging (fMRI) studies have revealed a reduction in activity within the S1 region in ACLR patients. Culiver et al. [54] demonstrated that, relative to healthy controls, ACLR patients exhibit significantly diminished activation in the S1 region during movements of both the affected and unaffected limbs. Criss et al. [50] compared S1 region activation during flexion and extension between the affected and unaffected limbs, revealing lower activation in the S1 region during activities involving the affected limb.

Proprioceptive signals detected by the ACL are conveyed via the spinal cord and relayed to the S1 region of the brain through the thalamus [57]. Following ACL injury, damage to the afferent pathways results in the loss of the ACL’s sensory function and diminished activation in the contralateral sensory cortex [44,58,59,60,61]. Current research indicates that proprioceptive function following ACL reconstruction surgery often fails to fully recover, and this deficit persists post-ACLR. Individuals compensate for reduced afferent input by upregulating other sensory processing mechanisms. This adaptation improves proprioceptive awareness, refines motor planning, optimizes muscle contraction strategies, and facilitates the achievement of dynamic joint stability. Although findings from EEG and fMRI studies appear contradictory, the observed discrepancies in current sensory cortex research outcomes likely arise from methodological variations. Specifically, EEG studies [29] have reported enhanced α-2 band desynchronization during weight-bearing tasks demanding proprioceptive compensation, whereas fMRI studies [54] have demonstrated decreased S1 activation levels during knee flexion control. This suggests that neural adaptation is task-dependent: proprioceptive challenges may amplify sensory processing, whereas simplified movements may unmask underlying deficits. Anatomically, the S1 region is adjacent to the M1 and the parieto-occipital junction. Furthermore, EEG scalp potential measurements possess low spatial resolution, rendering their results susceptible to contamination from activity in adjacent brain regions [61]. Notably, longitudinal research evidence [37] demonstrates that theta wave power exhibits a dynamic normalization trend during the 5–8 week and 12–16 week postoperative periods, underscoring the necessity for segmented temporal analysis.

#### 4.1.2. Increased Visual Cortex Activation

The visual center comprises the cuneus and lingual gyrus within the occipital lobe, situated adjacent to the calcarine sulcus. In alignment with prior research, ACLR patients demonstrate heightened activation in the visual center during simple post-surgical movements. An EEG study by An et al. [31] revealed that, relative to the healthy control group, ACLR patients exhibited elevated alpha-2 power in the primary visual cortex during the initial phase of single-leg standing. This suggests that ACLR patients may depend more heavily on internal cognitive processing for balance preparation and maintenance. Daghan Piskin’s study [39] demonstrated that during the execution of passing movements, ACLR players exhibited significantly diminished alpha wave desynchronization in the occipital lobe compared to healthy players. This likely results from ACLR individuals relying more heavily on visual cues during movement, thereby enhancing visual processing and manifesting more pronounced occipital lobe activity during motor tasks [35,44].

fMRI studies have also identified alterations in the activity of visual processing-related regions within ACLR populations. Criss et al. [44] observed that during the performance of combined hip–knee–ankle movements, ACLR patients demonstrated increased blood flow signals in regions associated with visual processing. Subsequently, Criss et al. [49] reported that during the execution of isolated knee flexion-extension movements, ACLR patients exhibited heightened activation in the lingual gyrus. Chaput et al. [45] documented that during the performance of balance tasks, ACLR patients demonstrated enhanced activation in the precuneus. These findings align with the earlier observations reported by Grooms et al. [19,60].

Following ACLR, patients exhibit heightened activation in visual-related regions during basic lower limb movements, indicative of an augmented reliance on visual feedback. This heightened activation in the visual center is likely attributable to the compromised mechanoreceptors subsequent to ACL injury, resulting in diminished signal transmission to the S1. In motor control, visual and proprioceptive inputs function as complementary sources of sensory information [62]. When proprioceptive input diminishes, individuals may depend more heavily on visual feedback during movement, given that visual adaptation to dynamically changing stimuli occurs more rapidly than the vestibular system’s response [63]. Research has demonstrated that with visual input, postural control in ACLR individuals is comparable to that observed in healthy individuals. Nevertheless, when visual input is compromised, their postural control undergoes significant deterioration [64,65,66].

#### 4.1.3. Diminished Activation Efficiency in the Motor Cortex

The motor center, situated within the prefrontal lobe of the brain, primarily comprises the primary motor cortex (M1), the supplementary motor area (SMA), and the premotor cortex (PMC). M1, situated in the precentral gyrus, is tasked with controlling motor commands and regulating posture and movement. During task execution, there is an increase in contralateral M1 activation.

Transcranial magnetic stimulation (TMS) studies have demonstrated a significant increase in the active motor threshold (AMT) of the surgical limb in ACLR patients [34,42,43]. Additionally, research has indicated that elevated AMT levels are not confined to the injured limb; quadriceps AMT levels in ACL reconstruction patients are also heightened [42,43,46]. Leung et al. [40] further compared corticospinal excitability between male and female ACLR patients, revealing that female patients exhibit lower excitability than their male counterparts.

Electroencephalography (EEG) studies have also identified diminished activation efficiency in the M1 among ACLR patients. The study by An et al. [31] found that compared to healthy controls, ACLR patients demonstrated elevated theta power in the contralateral M1 during the initial phase of single-leg standing. Sherman et al. [35] observed that during balance tasks, the ACLR group exhibited elevated α-2 and θ band powers in the bilateral motor cortices. Nyffenegger et al. [37] discovered that during joint angle reproduction tasks 5–8 weeks post-surgery, ACLR patients showed increased central θ band power in the unaffected limb. This suggests that ACLR patients necessitate greater neural activation when preparing for muscle contraction.

fMRI studies have demonstrated that during knee flexion and extension movements of the affected limb, ACLR patients exhibit heightened motor cortical activity. Schnittjer et al. [51] reported that in ACLR patients, the percentage change in the M1 signal during flexion and extension movements of the affected limb was significantly greater than that in the contralateral group, with the focus of parietal lobe activation being more posterior and superior. However, Culiver et al. [54] arrived at different conclusions, noting that compared to healthy individuals, the ACLR group demonstrated significantly reduced activation in the contralateral M1, S1, SMA, and precuneus during movements of both the affected and unaffected limbs. This discrepancy may be attributed to the study’s inclusion of patients in the early post-ACLR period (6.8 ± 1.2 weeks),who were still experiencing postoperative pain and swelling during activities. Furthermore, certain studies have compared brain activity during movements of the injured versus non-injured limbs. Criss et al. [50] demonstrated that during activities involving the non-injured limb, the activation levels of M1, PMC, SII, and SMA were higher compared to those of the injured side.

Sherman and colleagues conducted a series of studies employing Cortical–Muscle Coherence (CMC) to evaluate the impact of the motor cortex on muscle activity. The intensity of CMC reflects the extent of cortical drive to muscle activity; a higher CMC signifies improved synchronization between cortical and muscle activity, as well as a stronger cortical drive. In a 2023 study [34], Sherman observed that during a 50% maximal voluntary effort task, the γ-band CMC in the ACLR group was significantly lower compared to the control group, indicating a diminished cortical drive. In a subsequent study [38], it was demonstrated that within the ACLR group, reduced corticospinal excitability and activation levels in the sensory and motor cortices exhibited a weak correlation with smaller medial quadriceps muscle unit (MU) action potential amplitudes, a finding not present in the control group. Compared to the contralateral and control limbs, ACLR individuals exhibited earlier recruitment of larger MUs, along with reduced firing rates at both absolute and mass-normalized recruitment thresholds in their affected limbs. Riehm et al.’s [36] study revealed significant asymmetry in cortical muscle dynamics between the affected and unaffected legs within the ACLR group, a phenomenon not evident in the control group.

TMS research has demonstrated that ACLR patients exhibit elevated bilateral motor thresholds (AMT), signifying diminished corticospinal excitability. Unilateral injuries result in alterations in bilateral motor cortical excitability, suggesting that these injuries impact motor network reorganization beyond mere sensory and mechanical deficits. TMS indirectly assesses the functional state of the cortical motor system via external stimulation. Despite inherent limitations, the integration of TMS and fMRI studies has uncovered heightened contralateral M1 activation during basic knee flexion and extension movements, with asymmetry in CMC signifying diminished efficiency of motor cortical activation in ACLR patients. Diminished muscle information during action potentials necessitates a greater internal drive to elicit a response, resulting in diminished brain–muscle coordination. ACLR patients display neuromuscular coordination deficits, potentially attributable to sensory–motor integration disorders, corticospinal tract atrophy, and neural inhibition.

### 4.2. Cortical Activation Under Different States

#### 4.2.1. Characteristics of Cortical Activation During Motor Actions

Lehmann et al. [30] observed that during stance on the injured leg, ACLR patients demonstrated heightened alpha-2 band connectivity in the contralateral frontal–parietal, frontal–occipital, occipital–motor, and parietal–occipital regions, along with augmented ipsilateral occipital–motor and interhemispheric connections. The control group exhibited comparable trends across both legs. These heightened connections predominantly involved somatosensory and visual regions. Despite similar postural control abilities between the healthy control group and the ACLR group, ACLR patients may depend on additional neural resources for motor preparation and sensory feedback during initial single-leg stance to sustain stability. This could represent a compensatory mechanism employed by ACLR individuals to adapt to alterations in knee joint reconstruction and task complexity. Sherman et al. [35] demonstrated that under four single-limb balance task conditions, ACLR participants outperformed the control group in motor planning, sensory, and motor activities. In a follow-up study, Sherman et al. [33] observed that during the stimulus and response locking phases of a lower limb lateral choice reaction time task (Go/NoGo), the LRP area in ACLR patients was reduced compared to that in the control group. During the response selection and motor execution phases subsequent to stimulus presentation, ACLR patients exhibited diminished EEG amplitude, potentially indicating reduced brain engagement in motor preparation and execution, along with inhibitory processes in the response selection pathway. Giesche et al. [32] reported that during an unplanned landing task, ACLR participants exhibited marginally higher motor-related cortical potentials compared to healthy participants (small-to-moderate effect size). The brains of ACLR patients might necessitate heightened activity for both unconscious and conscious motor planning during jumping tasks. Piskin et al. [39] reported that during the execution of passing movements, source-derived event-related spectral perturbations revealed significant differences in posterior α (healthy players exhibited stronger α desynchronization, particularly between 1750 and 2250 ms) and frontal θ oscillations (ACLR players demonstrated more pronounced θ synchronization, notably between 750 and 1000 ms) between the two groups. The authors propose that ACLR players experience sensory-motor alterations during kicking, potentially requiring compensatory

Strategies like heightened attention, which may compromise visual–spatial information processing capabilities. The fNIRS study [55] demonstrated that during stair-climbing tasks, right-knee ACLR patients display significant negative activation in the ipsilateral premotor cortex (PMC), supplementary motor area (SMA), and primary somatosensory cortex (S1), a phenomenon not observed in healthy individuals. Despite patients’ self-reports not indicating significant knee joint dysfunction, the authors conclude that ACLR patients experience impairments in motor control strategies and sensory feedback mechanisms during the execution of repetitive complex motor tasks.

fMRI studies have demonstrated that the brain activation patterns of ACLR patients during motor tasks differ from those of healthy individuals. Specifically, during knee flexion-extension tasks, there is heightened activation in the parietal regions [44,51,54], which govern spatial orientation and perception; the prefrontal regions [19,42,50], which are crucial for motor planning; and the visual regions [50,51]. Furthermore, the interconnectivity among the prefrontal, parietal, and occipital lobes is markedly augmented [44].

Following ACLR, impaired knee joint afferent signals necessitate heightened attention and augmented motor planning for balance maintenance. This phenomenon results in augmented activation of brain regions governing planning and cognitive processing, alongside elevated functional connectivity among somatosensory, motor planning, and visual-related areas. The patients’ neural networks undergo substantial reorganization. Augmented inhibition in the motor cortex may indicate a cautious approach to movement, correlating with diminished motor control ability. Collectively, these alterations culminate in diminished balance control in ACLR patients, evidenced by heightened sway speed. ACLR patients demonstrate an inhibitory state in the neural processes of response selection and motor execution, yet necessitate additional planning for executing related actions. In alignment with prior research, ACLR patients necessitate heightened activation of motor and visual cortices during movement, potentially reallocating cognitive resources ordinarily dedicated to response selection and motor execution to complex task actions. Typically, individuals execute such simple actions with minimal cognitive engagement. Models of attention capacity suggest that cognitive load is finite [67]. If an action demands excessive cognitive resources, the availability of resources for response selection and execution diminishes. ACLR patients frequently compromise cognitive performance under heightened cognitive load to sustain adequate postural control. Research has demonstrated [68,69,70] that with escalating cognitive demands, the postural stability of ACLR patients diminishes.

#### 4.2.2. Characteristics of Cortical Activation During Resting Condition

Wang et al. [41] observed that in patients undergoing early postoperative anterior cruciate ligament reconstruction (ACLR), the amplitude of low-frequency fluctuations (ALFF) in the anterior cingulate gyrus and supplementary motor area significantly increased. Additionally, the fractional amplitude of low-frequency fluctuations (fALFF) in the right postcentral gyrus, inferior parietal lobule, superior marginal gyrus, anterior cingulate gyrus, and supplementary motor area also exhibited significant increases. At rest, significant differences in brain activation and network connectivity were observed between ACLR patients and healthy individuals, suggesting potential extensive neural reorganization in these patients. However, considering that Wang’s study primarily involved early postoperative patients and there were variations in participant types between the two studies, these differences might be influenced by alterations in brain activation due to postoperative swelling and pain.

#### 4.2.3. Cortical Activation Related to Emotion

Baez et al. [47] found that during a visual imagery task, ACLR patients exhibited changes in the cortical limbic system compared to healthy controls. Specifically, there was an increase in activity in the inferior parietal lobule and dorsal medial thalamus, along with a decrease in default network inhibition. Kim et al. [52] discovered that, when the ACLR group observed and imagined performing a vertical drop jump, brain activity in specific regions, including the cerebellum, amygdala, middle temporal gyrus, and temporal pole, positively correlated with Tampa Scale for Kinesiophobia-11 (TSK-11) scores. This correlation was not observed in the healthy population. During the observation, the activity of the right ventrolateral prefrontal cortex was lower in the ACLR group than in the healthy control group.

Despite undergoing ACLR surgery for an extended period, participants in both studies still exhibited changes in areas of the cortical limbic system while viewing specific execution images. This suggests that they may be more susceptible to fear, anxiety, and pain responses related to movement tasks and daily activities. These changes may result from memories of ACL injury, postoperative recovery, and an excessive focus on potential harm. ACLR patients may also experience movement-related fear during task execution, potentially linked to prolonged processing times for movement planning or perceptual judgment. Emotion-related patterns of brain activation can impact neuromuscular control of the knee joint. For example, during vertical drop jumps, ACLR patients exhibit stiffer landing strategies [71], which may increase the risk of secondary ACL injuries.

### 4.3. Brain Structural Changes

Among the reviewed studies, three examined alterations in gray and white matter volumes in ACLR patients. Lepley et al. [43] quantified hemispheric discrepancies in the corticospinal tract structure of ACLR patients, revealing a smaller volume in the contralateral hemisphere compared to the uninjured side, indicative of potential corticospinal tract atrophy in the affected limb. Flanagan et al. [46] observed that female ACLR patients exhibited diminished cortical thickness in the sensorimotor cortex (PMC), especially in the paracentral lobule (PCL), relative to healthy individuals, during rest. Furthermore, within the topological structure of the cortical motor network, the significance of non-limb motor areas augmented, whereas the efficacy and centripetal impact of the primary somatosensory area diminished. Yu et al. [53] reported compromised integrity of the corticospinal tract (CST) in the ACLR group, evidenced by lower fractional anisotropy (FA) and higher radial diffusivity (RD), with these impairments moderately correlating with the post-surgery duration (extended postoperative time and elevated RD). Lepley et al. [43] reported elevated mean diffusivity (MD) values in the corticospinal tract (CST) of the injured limb hemisphere in ACLR patients, indicative of increased water molecule diffusion within white matter fiber bundles, whereas Yu et al. [53] observed no interhemispheric differences in MD values. This discrepancy might arise from Lepley et al.’s omission of a healthy control group for detecting variations in affected CNS structures relative to ACLR patients, coupled with age disparities among participants (those in Yu et al.’s 2025 study [53] being roughly a decade older than Lepley’s cohort), potentially influencing the neuroplasticity of MD.

Animal studies [72] have shown direct neural connections between the ACL, spinal cord, brainstem, and cerebellum. Proprioceptive motor signals from the lower limbs ascend through the spinocerebellar tract to the ipsilateral cerebellum, where they are integrated. The cerebellum regulates movement accuracy by modulating the excitability of its internal and spinal motor nuclei. Neural signals initiating voluntary movement originate in the cerebral cortex and are transmitted to the cerebellum via the corticobulbar and corticospinal tracts, with synapses occurring in the pons. Once excited, the cerebellar cortex relays feedback neural impulses through the dentate nucleus and thalamus to the PMC and M1 in the brain. After ACL injury, the ascending sensory pathways from the ligament become disrupted, delayed, or altered, leading to reduced activation of the injured side of the cerebellum [73]. Concurrently, activation in the contralateral corticospinal tract and sensorimotor areas of the brain decreases. These changes may worsen over time, particularly with reduced motor output to protect the injured side, ultimately leading to structural changes, such as volume loss in these regions. In the studies by Lepley et al. [43] and Flanagan et al. [46], changes in central morphology above the spinal cord were observed, indicating that central nervous system alterations in ACL injury patients do not return to normal due to surgical reconstruction or the passage of time.

### 4.4. Changes in Neural Activity Patterns After ACLR and Their Implications

#### 4.4.1. The Impact of Postoperative Time and Graft Type on Neural Activation Patterns

The neural adaptation process following ACLR is characterized by distinct stages of cortical remodeling. Studies focusing on patients in the early postoperative phase (<3 months) have identified distinct neural activation characteristics. Wang et al. [41] observed that, during rest between 2 and 12 weeks post-surgery, ALFF/fALFF values significantly increased in sensory integration regions (bilateral mid-cingulate cortex and supplementary motor area), suggesting the potential presence of an acute compensatory mechanism. Conversely, Culiver et al. [54] recorded a significant reduction in activation of the ipsilateral M1, S1, and SMA during active knee flexion and extension at 6.8 ± 1.2 weeks post-surgery, potentially linked to inhibition caused by immediate postoperative edema or pain. Nyffenegger et al. [37] provided longitudinal evidence: compared to the 12–16 week postoperative period, theta wave power was significantly higher (*p* < 0.05) during joint position sense testing in the unaffected limb at 5–8 weeks post-surgery, indicating that sensorimotor processing may be dynamically returning to normal.

In the late postoperative phase (>6 months), studies consistently reported a decline in motor efficiency. Sherman et al. [34] observed a decrease in cortico-muscular gamma-band coherence within the ACLR group, specifically in both the vastus medialis and vastus lateralis, during force control tasks; meanwhile, Criss et al. [50] observed that S1 activation was lower in the injured limb compared to the uninjured side during movement. Notably, Yu et al. [53] found evidence of progressive white matter degeneration: the radial diffusivity (RD) of the corticospinal tract correlated positively with postoperative time (after adjusting for age/BMI, r = 0.623, *p* < 0.001), suggesting potential chronic microstructural deterioration.

Regarding graft type, due to heterogeneity in the reported studies—including that 8 out of 27 did not clearly specify the graft type used—the current evidence is insufficient to draw definitive conclusions. This heterogeneity highlights a critical research gap: currently, no studies have systematically analyzed the modulatory effects of different graft choices (such as hamstring tendon, bone-patellar tendon-bone, or quadriceps tendon) on long-term cortical remodeling, necessitating dedicated comparative research.

#### 4.4.2. The Association Between Brain Activity Alterations and Clinical Functional Outcomes

The disparities in brain activation between ACLR patients and healthy individuals likely arise from adaptive modifications the body undergoes following ACL rupture, closely tied to the patients’ clinical functionalities. Criss et al. [50] demonstrated a positive correlation between activity in the ipsilateral SII and SMA regions and IKDC scores. Grooms et al. [19] reported that individuals exhibiting reduced SMA activation levels subsequently incurred non-contact ACL injuries on the unaffected side. Chaput et al. [45] observed that in the ACLR group, visual cognition correlated with heightened neural activity in the precuneus and posterior cingulate cortex, a relationship absent in healthy individuals. Furthermore, visual cognitive abilities in the ACLR group exhibited a negative correlation with proprioceptive errors and landing stability time. Strong et al. [48] revealed that during the JPS task, activation in the insula, anterior cingulate, and angular gyrus within the ACLR group positively correlated with error rates.

In the pioneering study by Courtney et al. [12], participants with varying functional outcomes post-ACLR displayed unique patterns of brain activation. He classified all ACL reconstruction participants into three groups based on their functional recovery levels: the highest-functioning Copers group, characterized by no strength deficits and full recovery of movements, including cutting and pivoting; the moderately functioning Adapters group, with no strength deficits or recent “giving way” incidents, and partial movement recovery; and the lowest-functioning non-Copers group, exhibiting strength deficits, frequent “giving way” incidents, and an inability to recover movements. Among these groups, the Copers group displayed proprioceptive deficits, absence of P27 potentials, and premature activation of the biceps femoris; the Adapters group exhibited normal proprioception and P27 potentials, coupled with early activation of the gastrocnemius, whereas the non-Copers group showed proprioceptive deficits, normal P27 potentials, and a lack of coordinated muscle activation patterns. The Copers group demonstrated the most adaptive changes, potentially reflecting the central nervous system’s adjustment to ACL reconstruction. Conversely, despite retaining proprioception, the Adapters group’s limited central nervous system adjustments impeded the full recovery of motor function. The non-Copers group likely exhibited poor functional performance due to the central nervous system’s failure to make adaptive adjustments to ACL injury. In prospective studies conducted by Chaput et al. [45] and Diekfuss et al. [74], individuals with diminished connectivity in sensory and motor regions subsequently experienced ACL injuries.

This study utilizes a range of technical methodologies to assess the correlation between alterations in brain activity and clinical outcomes post-ACL reconstruction, uncovering disparities in cortical activation strategies between ACLR patients and healthy controls, as well as heterogeneity in cortical activation strategies within the ACLR cohort. The activation of sensory integration regions is intimately linked to knee joint functionality. Individuals with superior knee joint function may demonstrate enhanced proprioceptive integration capabilities relative to those with diminished function, who might lack adaptive neural adjustments post-ACL injury. Additionally, superior performance in activities among ACLR patients correlates with the engagement of multiple brain regions to augment visual cognitive functions, indicating that the disruption of afferent signals post-ACLR may result in diminished proprioception and stability. To mitigate this deficit, individuals post-ACLR may depend on visual cognitive functions (e.g., visual memory and visuomotor skills) to preserve proprioception and dynamic stability.

### 4.5. Limitations and Future Directions

This review identified that methodological, outcome, and clinical heterogeneity hinders the conduct of a meta-analysis. Although narrative synthesis can provide a comprehensive overview, it is unable to quantify effect sizes or establish dose–response relationships. Key contributing factors include: heterogeneity in neuroimaging techniques—where EEG records oscillatory activity, TMS assesses corticospinal excitability, and fMRI and fNIRS reflect hemodynamic responses, with each technique revealing different dimensions of neuroplasticity; heterogeneity in task paradigms—where different motor tasks activate distinct neural circuits, thereby limiting the generalizability of the findings; and patient-related factors—where unreported graft types and postoperative time points may confound the interpretation of neural adaptation patterns. Given the diversity in research methodologies and the presence of confounding factors, the existing body of research is insufficient to comprehensively elucidate the mechanisms and impacts of brain plasticity following ACLR. Therefore, future research that achieves consensus on core outcome measures and stratifies findings by graft type and postoperative stage will help enhance comparability across studies.

Neuroimaging studies frequently encounter challenges related to variability and false-positive outcomes. To mitigate potential errors, it is essential to regulate factors that may contribute to variability. Gender and age substantially impact brain activation [74]; among the incorporated studies, only one investigation examined sex-specific subgroups [40], revealing a discernible divergence in neurokinetic drive between male and female ACLR patients. Given the documented sex-specific predilection in ACL injury susceptibility [75] and established neurophysiological distinctions in cortical activation and neuromuscular regulation across sexes [76], we advocate for the systematic integration of gender as a critical analytical variable in subsequent research. The majority of participants (aged 20–30 years) represented a restricted demographic spectrum, with merely two studies enrolling subjects exceeding 30 years of age [41,53]. We acknowledge this as a methodological limitation and recommend future investigations incorporate cohorts exhibiting broader age distributions. Given that central reorganization post-unilateral ACLR extends beyond the injured side, the practice of using the uninjured side as a comparative control necessitates re-assessment. Future studies should strive to select control groups that are matched across multiple dimensions, including age, gender, height, weight, historical and current activity levels, types of sports participation, educational background, and dominant side, to ensure accurate limb-to-limb comparisons.

Current neuroimaging investigations assessing cerebral reorganization frequently suffer from methodological constraints arising from restricted participant mobility, thereby compromising the ecological validity of research outcomes. The majority of the included studies (20 of 27) employed simplified laboratory paradigms—such as isometric contractions, single-leg stance, or constrained knee flexion-extension (0–45°)—that fail to replicate the multi-joint coordination, decision-making demands, and unpredictable environments inherent in athletic activities. This methodological limitation may account for the observed discrepancy wherein patients exhibit adequate functional performance in controlled settings yet remain susceptible to re-injury during sport-specific maneuvers. For instance, Cao et al. [55] documented aberrant premotor cortex activation during stair negotiation (a functional task), whereas simplified knee movements in fMRI investigations [54] revealed inconsistent motor cortex activation patterns. Future research should prioritize dynamic sport-relevant paradigms, such as integrating virtual reality (VR) systems with neuroimaging or utilizing fMRI-compatible motion simulators, to better capture neuroadaptive alterations occurring during complex activities like cutting maneuvers or landing.

### 4.6. Recommendations in Rehabilitation Therapy

Targeting the identified neural mechanisms through intervention is anticipated to optimize rehabilitation outcomes.

Quadriceps electrical stimulation, a technique rooted in sensory enhancement therapy [77,78], can improve sensory processing function in the S1. This intervention may promote neuroplasticity and thereby remodel knee joint proprioception [54,79]. TMS targeting the M1 combined with MVC can effectively enhance corticospinal conduction efficiency and improve motor coordination [80,81]. Combining balance training with flicker glasses intervention can optimize sensory–visual integration and reduce excessive activation associated with visual dependence in the occipital cortex [25,77,82]. Cognitive–motor integration training can utilize external focus motor learning strategies, such as verbal guidance and biofeedback [83], or dual-task training [69]. These strategies aim to shift motor control from cortical to subcortical dominance, thereby enhancing movement automation. Additionally, it is also necessary to monitor changes in patients’ psychological and cognitive status. During the rehabilitation process, it is important to focus on training mental health and cognitive function and employ multi-dimensional approaches to enhance rehabilitation outcomes. Verifying the actual effectiveness of these neuroregulation-based rehabilitation strategies is crucial and requires future clinical research.

## 5. Conclusions

This systematic review examined 27 studies focusing on the characteristics of brain activity and structure post-ACLR, aiming to synthesize the observed abnormalities in brain activity within the ACLR population and their implications for patient functional outcomes. Current research demonstrates widespread disparities in brain activity between ACLR patients and healthy controls, with these disparities being strongly associated with lower limb functionality. The specific manifestations are as follows:

(1) Alterations in sensory cortex activation, augmented visual cortex activation, and diminished efficiency of motor cortex activation: Patients exhibit an increased dependence on visual input for motor control during task execution.
(2) Modifications in neural network connectivity: Augmented connectivity among the prefrontal, parietal, and occipital lobes, yet diminished cognitive–motor efficiency. Attenuated inhibitory function of the emotion-related default network, coupled with heightened activation in limbic system regions.
(3) Diminished volume of the corticospinal tract corresponding to the injured limb and reduced thickness of the sensorimotor cortex: These structural alterations further compromise the patient’s motor function.
(4) Augmented activation in sensory integration areas and visual cognition-related regions among ACLR patients, correlated with enhanced lower limb function: This suggests that compensatory alterations in brain activation post-ACLR partially facilitate functional recovery.

In summary, the distinct brain activation patterns in ACL reconstruction patients substantially influence lower limb functionality. Future research and rehabilitation protocols must comprehensively account for these alterations to enhance rehabilitation efficacy.

## Figures and Tables

**Table 1:** Methodological characteristics and main results of studies included in the analysis.

Research	Participant Information				Technical Specifications	Test Task	Results
	Group/*n* (Male/Female)	Age (Years)	Injury/Postoperative Duration	Injury/Surgery Type			
Test Technique: EEG (*n* = 11)							
An et al. (2019) [29]	ACLR group: 17 participants (No information); healthy controls group: 17 participants	22.29 ± 3.77 26.94 ± 5.56	3.48 ± 2.06 years postoperative	No information	EEG spectral characteristics	Degree of relaxation in the three-dimensional spatial vertical translation of the knee	Compared with the healthy limb and matched limb of the control group, ACLR participants showed increased cortical activity (higher alpha-2 ERD) in the reconstructed limb during early weight-bearing (ERD1) phases, although there were no significant group differences in joint relaxation. During late joint load phases, ACLR participants demonstrated greater somatosensory cortical activity (ERD3) relative to the control group, without differences in relaxation.
Lehmann et al. (2021) [30]	ACLR group: 12 participants (7/5); healthy controls group: 12 participants (7/5)	25.1 ± 3.2 25.5 ± 3.8	44.4 ± 4.5 days postoperative	5 right knees 7 left knees HT grafts (4 cases with meniscal repair, 6 cases with secondary injury)	EEG spectral characteristics	Ten 30 s trials of single-leg standing	In the ACLR group, functional connectivity in the alpha-2 frequency band significantly increased while standing on the injured leg, involving frontal–parietal, frontal–occipital, occipital–motor, occipital–parietal, and parietal–occipital connections in the contralateral hemisphere; occipital–motor connections in the ipsilateral hemisphere; and interhemispheric frontal–frontal, occipital–motor, and occipital–parietal connections. The control group showed a similar trend in both legs.
An et al. (2022) [31]	ACLR group:15 participants (10/5). healthy control group: 17 participants (10/5)	23.13 ± 3.20 23.07 ± 3.45	2.97 ± 2.28 years postoperative	No information	EEG spectral characteristics	During single-leg stance tasks on stable and unstable platforms (moving in all directions by 15°), the supporting knee was flexed at 5° and the non-supporting knee at 45° (only for the reconstructed or matched limb, not the healthy limb), with visual feedback on the center of pressure.	During single-leg stance, ACLR participants displayed higher theta power in the contralateral primary motor cortex, higher alpha-2 power in the ipsilateral prefrontal cortex, lower alpha-2 power in the central somatosensory cortex, and increased alpha-2 power in the primary visual cortex.
Giesche et al. (2022) [32]	ACLR group: 10 men; healthy control group: 17 men.	28 ± 4 28 ± 4	Postoperative period: 63 ± 35 months.	No information Any type of graft, including those with meniscal damage	EEG spectral characteristics	43 ± 4 planned (landing leg displayed before takeoff) and 51 ± 5 unplanned (visual cue during flight) reactive jumps with single-leg landings.	ACLR participants showed slightly higher MRCP values in unplanned tasks, with moderate effect size.
Sherman et al. (2023b) [33]	ACLR group: 20 participants (8/12); healthy control group: 20 participants (8/12).	21.2 ± 2.2 21.5 ± 2.2	Postoperative period: 15.1 ± 9.6 months.	No information 10 BTB, 5 HT, 4 QT, 1 Allo	LRP/AMT	Lateralized choice reaction time task in a seated position. Based on the position of the ball and the color of the jersey on the screen, participants quickly kicked the ball with their left or right foot. EEG was used to record brain activity.	In the ACLR group, the stimulus-locked LRP area was smaller than that of the control group under both Go and NoGo conditions, indicating lower EEG activity amplitude in the response selection phase after stimulus onset. The ACLR group also had significantly higher AMT than the control group.
Sherman et al. (2023c) [34]	ACLR group: 20 participants (8/12); healthy control group: 20 participants (8/12).	21.2 ± 2.2 21.5 ± 2.2	Postoperative period: 15.1 ± 9.6 months.	No information 10 BTB, 5 HT, 4 QT, 1 Allo	Contralateral motor cortex and ipsilateral quadriceps CMC.	Knee extension torque control task at 50% MVC with 8 trapezoidal waveform traces.	The γ-band CMC was significantly lower in the ACLR group compared to the control group (vastus medialis: d = 0.8; vastus lateralis: d = 0.7), and the active motor threshold was higher (d = 1.0), indicating reduced cortical drive.
Sherman et al. (2023a) [35]	ACLR group: 20 participants (8/12); healthy control group: 20 participants (8/12).	21.2 ± 2.2 21.5 ± 2.2	Postoperative period: 15.1 ± 9.6 months.	No information 10 BTB, 5 HT, 4 QT, 1 Allo	EEG spectral characteristics.	Single-limb balance tasks were performed under four conditions: internal focus (IF), object-based external focus (EF), goal-based external focus (EF), and transcutaneous electrical nerve stimulation (TENS).	Under all conditions, parietal α-2 frequency power was higher, as was bilateral motor cortex α-2 frequency power, along with θ frequency power.
Riehm et al. (2024) [36]	ACLR group: 20 participants (8/12); healthy control group: 20 participants (8/12).	21.2 ± 2.2 21.5 ± 2.2	Postoperative period: 15.1 ± 9.6 months.	No information 10 BTB, 5 HT, 4 QT, 1 Allo	CM-cRQA	Knee extension torque control task at 50% maximal voluntary contraction (MVC) with 8 trapezoidal waveform traces.	Significant asymmetry in cortico-muscular dynamics was observed between the affected and unaffected legs in the ACLR group, whereas the control group showed no such asymmetry in cortico-muscular dynamics.
Nyffenegger et al. (2025) [37]	ACLR group: 12 participants (9/3); healthy control group: 12 participants (9/3).	25.3 ± 6.4 28.8 ± 9.7	First test (M1): 5–8 weeks after surgery Second test (M2): 12–16 weeks after surgery	4 participants injured dominant leg 1HT,11QT	EEG spectral characteristics.	Active knee Joint Position Sense (JPS) tests were performed in the open kinetic chain, starting with a knee flexion angle of 90° and targeting an angle of 50°	Participants with ACLR exhibited significantly higher central θ power during JPS testing with their uninvolved leg at M1 compared with M2.
Sherman et al. (2025) [38]	ACLR group: 20 participants (8 males/12 females); healthy control group: 20 participants (8 males/12 females).	21.2 ± 2.2 21.5 ± 2.2	Postoperative period: 15.1 ± 9.6 months.	No information 10 BTB, 5 HT, 4 QT, 1 Allo	CM-cRQA	Knee extension torque control task at 50% maximal voluntary contraction (MVC) with 8 trapezoidal waveform traces.	Participants with ACLR had lower corticospinal excitability and lower contralateral hemisphere motor cortex activations during quadriceps contractions. Lower corticospinal excitability and lower activations in the sensory and motor cortices were weakly associated with smaller MU action potential amplitudes, whereas group was not.
Piskin et al. (2025) [39]	ACLR group: 10 players (6 males/4 females); healthy control group: 15 players (9 males/6 females).	25.5 ± 3.0 20.5 ± 4.5	Postoperative period:5.05 ± 2.6 months.	No information 3 BTB, 6 HT, 1 QT	EEG (ERSP)	short-distance passing motion	The ACLR group exhibited significantly higher entropy values in foot external rotation. Source-derived event-related spectral perturbations indicated significant differences in posterior α (healthy players showed stronger α desynchronization, with significant differences observed between 1750 and 2250 ms) and frontal θ oscillations (ACLR players had more pronounced θ synchronization, with significant differences observed between 750 and 1000 ms) between the two groups.
Testing technique: TMS. (*n* = 1)							
Leung et al. (2024) [40]	ACL injury group: 20 participants (9 males/11 females)h healthy control group: 18 participants (9 males/11 females).	23.9 ± 6.3 23.7 ± 5.9	Postoperative period: 5.4 ± 1.1 months.	No information 14 BTB, 2 HT, 1 QT, 3 Allo	AMT assessment of the CSE	Participants were seated with the hip to 90° and the knee to 60° to perform 3 MVIC, each lasting 5 s.	No significant between group differences in AMT on either side (*p* ≥ 0.395). There were significant between-group differences in the slope of the stimulus-response curve of the surgical side (*p* = 0.007),
Testing technique: MRI (*n* = 14)							
Wang et al. (2019) [41]	ACLR group: 18 participants (10 males/8 females); healthy control group: 17 participants (10 males/7 females).	36 ± 10 35 ± 11	Postoperative period: 2–12 weeks.	Left knee ACL reconstruction with autograft preservation.	rs-fMRI	Eyes closed, staying alert, avoiding active mental activity.	The ACLR group showed higher ALFF in bilateral middle cingulate gyri, involving the supplementary motor area. fALFF activation cluster 1 was higher in the right postcentral gyrus, involving the right inferior parietal lobule and right supramarginal gyrus; activation cluster 2 was higher in the right middle cingulate gyrus, involving the right supplementary motor area.
Lepley et al. (2019) [42]	ACLR group: 11 participants (5 males/6 females); healthy control group: 11 participants (5 males/6 females).	22.6 ± 1.8 23.2 ± 1.6	Postoperative period: 69.4 ± 22.4 months.	No information 9 BTB grafts 2 HT grafts.	fMRI/AMT	Independent cyclic flexion-extension movement of the right knee from 0° to 45°; MEP amplitude induced at 120% AMT.	The ACLR group showed higher activation in the gyrus rectus, inferior frontal pole, paracingulate gyrus, and anterior cingulate gyrus. The AMT was higher bilaterally in the ACLR group, and MEP was smaller in the injured limb.
Lepley et al. (2020) [43]	ACLR group: 10 participants (4 males/6 females), comparing injured and uninjured sides.	22.6 ± 1.9	Postoperative period: 70.0 ± 23.6 months (66.6–96.5 months).	No information.	DTI/MEP	DTI was used to scan the structural characteristics of the corticospinal tract. At 120% AMT intensity, 5 MEPs were induced, and the peak-to-peak MEP amplitude average was calculated.	Compared to the uninjured limb, the hemisphere of the ACLR-injured limb showed smaller corticospinal tract volume, lower FA values, and higher MD values, indicating white matter damage and reduced corticospinal tract excitability. Corticospinal tract volume was significantly positively correlated with excitability.
Criss et al. (2020) [44]	ACLR group: 15 participants (7 males/8 females); healthy control group: 15 participants (7 males/8 females).	20.94 ± 2.68 22.53 ± 2.47	Postoperative period: 43.33 ± 33.14 months.	Left knees 14 HT grafts, 1 BTB grafts.	fMRI	Unilateral left knee (ACLR knee or matched healthy control knee) cyclic knee and hip extension/flexion (similar to heel slides).	The ACL group showed increased BOLD signal in the superior parietal lobe and left fusiform gyrus in the occipital lobe and visual pathway, as well as in the left lateral occipital cortex, angular gyrus, and parietal lobe.
Chaput et al. (2022) [45]	ACLR group: 16 participants (6 males/10 females); healthy control group: 15 participants (6 males/9 females).	21.5 ± 2.6 2.9 ± 3.03	Postoperative period: 41.4 ± 33.0 months.	Left knees 13 HT grafts, 3 BTB grafts.	fMRI	Independent cyclic flexion-extension movement of the right knee from 0° to 45°.	Visual cognition was associated with increased neural activity in the precuneus and posterior cingulate cortex in the ACLR group, but not in the control group. Visual memory and visuomotor abilities were negatively correlated with proprioceptive error and landing stability time.
Flanagan et al. (2021) [46]	ACLR group: 9 female participants; healthy control group: 11 female participants.	20.6 ± 1.4 20.6 ± 2.3	3.2 ± 1.1 years.	5 left knees and 6 right knees; 5 HT grafts and 4 BTB grafts.	MRI (DWI)	Resting-state scanning.	Cortical thickness was reduced in the somatosensory cortex, particularly in the paracentral lobule (PCL), in the ACLR group. The topological structure of the cortical motor network changed in the ACLR group, with increased importance in non-limb motor areas and decreased effectiveness and eccentricity in primary somatosensory regions.
Baez et al. (2021) [47]	ACLR group: 12 female participants; healthy control group: 12 female participants.	21.5 ± 6.8 23.0 ± 1.8	Duration: 5.5 ± 4.2 years.	Left knee No information.	fMRI	Motor imagery and daily life activity imagery tasks were conducted.	In the ACLR group, the following changes were observed in the cortical-limbic system regions during imagery tasks: Increased activity in the inferior parietal lobule and dorsomedial thalamus. Decreased inhibition of the default mode network, with an inability to suppress the default mode network, including the posterior cingulate cortex, precuneus, and ventromedial prefrontal cortex. Task-specific changes: Increased activity in the cerebellum and inferior occipital cortex when imagining motor-related images.
Strong et al. (2022) [48]	ACLR group: 21 participants (8 males/13 females); healthy control group: 19 participants(7 males/12 females).	Right knee injury (24.8 ± 4.2); left knee (28.2 ± 4.7). Healthy participants 27.1 ± 4.6	Average postoperative period: 23 months.	10 right knee (4 males/6 females, 20.0 ± 9.7 months); left knee, 11 cases (4 males/7 females, 28.5 ± 18.6 months); HT grafts.	fMRI	Knee joint position sense (JPS) testing was performed during functional magnetic resonance imaging (fMRI).	Both groups activated proprioception-related brain regions, such as the somatosensory cortex, prefrontal cortex, and insula, during the JPS test. However, no significant differences were found in brain responses between the groups. JPS error was positively correlated with activation in the contralateral insula, anterior cingulate cortex, and supramarginal gyrus.
Criss et al. (2023) [49]	ACLR group: 22 participants (8 males/14 females); healthy control group: 22 participants (8 males/14 females).	22.1 ± 2.6 22.9 ± 2.7	Postoperative period: 4.6 ± 2.6 years.	7 right knees and 15 left knees. No information.	fMRI	Independent cyclic flexion-extension movement of the right knee from 0° to 45°.	In the ACLR group, increased activation was observed in the lingual gyrus, contralateral premotor cortex, and contralateral parietal lobe.
Criss et al. (2023) [50]	ACLR group: 25 participants (10 males/15 females), comparing injured and uninjured sides.	21.8 ± 2.6	Postoperative period: 51.6 ± 32.9 months.	7 right knees and 18 left knees; 15 HT grafts 10 BTB grafts.	fMRI	Independent cyclic flexion-extension movement of the right knee from 0° to 45°.	Compared to the injured side, the uninjured side showed higher activation in the primary motor, primary somatosensory, premotor, secondary somatosensory cortices, supplementary motor area (SMA), and cerebellum.
Schnittjer et al. (2023) [51]	ACLR group: 18 participants (8 males/10 females); healthy control group: 18 participants (7 males/11 females).	21.00 ± 5.25 22.00 ± 4.00	Postoperative period: 45.94 ± 7.55 months.	Left knee (No information).	fMRI	Independent cyclic flexion-extension movement of the right knee from 0° to 45°.	During movement of the affected limb in the ACLR group, motor cortex activity increased, activating unique parietal regions, including the inferior parietal lobule and lateral occipital cortex (upper region). The peak voxel position in the frontal and parietal cortices was more medial and superior, while the parietal peak voxel position was more posterior and superior. Cerebellar activity was more focused.
Kim et al. (2023) [52]	ACLR group: 13 participants (5 males/8 females); healthy control group: 13 participants (5 males/8 females).	20.62 ± 1.93 22.92 ± 3.17	Postoperative period: 1–10 years.	No information.	fMRI	Action observation drop vertical jump (AO-DVJ) paradigm; participants observed and imagined performing a drop vertical jump.	In the ACLR group, right ventrolateral prefrontal cortex activity was lower. Brain activity in certain cerebellar regions, amygdala, middle temporal gyrus, and temporal pole positively correlated with TSK-11 scores. No correlation between brain activity and TSK-11 scores was found in the control group.
Yu et al. (2025) [53]	ACLR group: 26 participants (23 males/3 females); healthy control group: 26 participants (22 males/4 females).	36.35 ± 6.39 32.85 ± 9.20	Postoperative period:15.0 months (interquartile range = 12.0–19.5 months)	14 right knees and 12 left knees No information.	DTI assessment of the CST structure.	Resting-state scanning.	ACLR had moderately lower fractional anisotropy (Cohen d = −0.666; 95% CI = −1.221, −0.104; *p* = 0.01), lower axial diffusivity (Cohen d = −0.526; 95% CI = −1.077, 0.030; *p* = 0.03), higher radial diffusivity (RD; Cohen d = 0.514; 95% CI = −0.042, 1.064; *p* = 0.04), and smaller Y-Balance Test anterior-reach distance (Cohen d = −0.743; 95% CI = −1.302, −0.177; *p* = 0.005) compared with healthy controls. The RD values were correlated with the postoperative duration (r = 0.623, *p* < 0.001) after controlling for age, sex, and body mass index in patients with ACLR.
Culiver et al. (2025) [54]	ACLR group: 15 participants (7 males/8 females); healthy control group: 15 participants (7 males/8 females)	21.9 ± 4.3 23.1 ± 3.1	Postoperative period:6.8 ± 1.2 weeks	4 Right knees 11 left knees 10 BTB grafts 5 HT grafts.	fMRI	Independent cyclic flexion-extension movement of the right knee from 0° to 45°.	Compared with the control group, during movement of the ACLR-involved and uninvolved knees, there was a significant reduction in the following regions: ipsilateral M1, ipsilateral/contra-lateral S1, SMA, and precuneus (*p* < 0.05, effect size 0.5–1.4).
Testing technique: fnirs(*n* = 1)							
Cao et al. (2025) [55]	ACLR group: 27 participants (23 males/4 females); healthy control group: 15 participants (23 males/4 females)	25.6 ± 2.3 25.8 ± 2.8	Postoperative period:4.6 ± 1.4 months	Right knees No information.	fNIRS	Repeat the upstairs task. Go up the stairs for 15 s. Then, after a 20 s rest, repeat the upstairs task for 15 s; a total of 4 sets were completed.	The ACLR group showed a significant decrease in channel 25β values (corresponding to the premotor and supplementary motor cortices), while the healthy control group had significantly higher β values in channel 33 (primary somatosensory cortex)

ERSP: event-related spectral perturbation; MVIC: maximum voluntary isometric; CST: corticospinal tract; CSE: corticospinal excitability; CM-cRQA: Cortico-Muscular cross-Recurrence Quantification Analysis; LRP: lateralized readiness potential definition: an EEG component reflecting motor preparation and response selection in choice-reaction tasks; BTB: bone-patellar tendon-bone; HT: hamstring tendon; QT: quadriceps tendon; Allo: tendon tissue from a donor.

**Table 2:** Prevalence of key neural alterations in ACLR patients (*n* = 27 studies).

Neural Alteration	Number of Studies	Primary Techniques	Key Clinical Correlations
Sensory Cortex Changes	4	EEG (2), fMRI (2)	• Enhanced proprioceptive awareness during weight-bearing [29] • Reduced activation during knee flexion [54]
Increased visual cortex activation	5	EEG (1), fMRI (4)	• Greater dependence on visual feedback for balance [31] • Correlated with reduced postural stability in visually deprived conditions [44]
Reduced Motor Cortex Efficiency	8	TMS (3), EEG (3), fMRI (2)	• Elevated AMT linked to quadriceps weakness [42] • Lower γ-band CMC associated with reduced cortical drive [34]
Fronto-Parietal-Occipital Connectivity	1	EEG (1)	• Compensatory mechanism for postural control [30]
Default Mode Network Inhibition	1	fMRI (1)	• Inability to suppress DMN during motor imagery [47]
Limbic System Activation	2	fMRI (2)	• Positive correlation with TSK-11 scores (fear of movement) [52]
Corticospinal Tract Atrophy	2	DTI (2)	• Reduced volume correlated with lower excitability [43] • RD values increased with postoperative duration [53]
Sensorimotor Cortex Thinning	1	MRI-DWI (1)	• Altered motor network topology [46]
Sensory/Visual Integration and Function	2	fMRI (2)	• Positive correlation with IKDC scores [50] • Negative correlation with proprioceptive error [45]

EEG: electroencephalography; fMRI: functional magnetic resonance imaging; TMS: transcranial magnetic stimulation; MRI-DWI: Magnetic Resonance Imaging-Diffusion Weighted Imaging; DTI: diffusion tensor imaging; AMT: active motor threshold; CMC: Cortical–Muscle Coherence; IKDC: International Knee Documentation Committee; DMN: default mode network; RD: Radial Diffusion; TSK-11: Tampa Scale for Kinesiophobia-11.

## Data Availability

The original contributions presented in this study are included in the article and Supplementary Materials. Further inquiries can be directed to the corresponding author.

## Supplementary Materials

The following supporting information can be downloaded at: https://www.mdpi.com/article/10.3390/brainsci15080831/s1, Figure S1: PRISMA flowchart; Table S1: PRISMA checklist; Table S2: Risk of bias assessment details. Ref. [84] is cited in Supplementary Materials.
