# Supplementary material for: Advances in Research on Brain Structure and Activation Characteristics in Patients with Anterior Cruciate Ligament Reconstruction: A Systematic Review
Source: Brain Sci. 2025 Aug 1;15(8):831. doi: 10.3390/brainsci15080831 (PMC12384111; doi:10.3390/brainsci15080831)
Supplement: Supplementary file 1 [file brainsci-15-00831-s001.zip › Supplementary Figure S1.pdf]

PRISMA 2020 flow diagram for new systematic reviews which included searches of databases and registers only

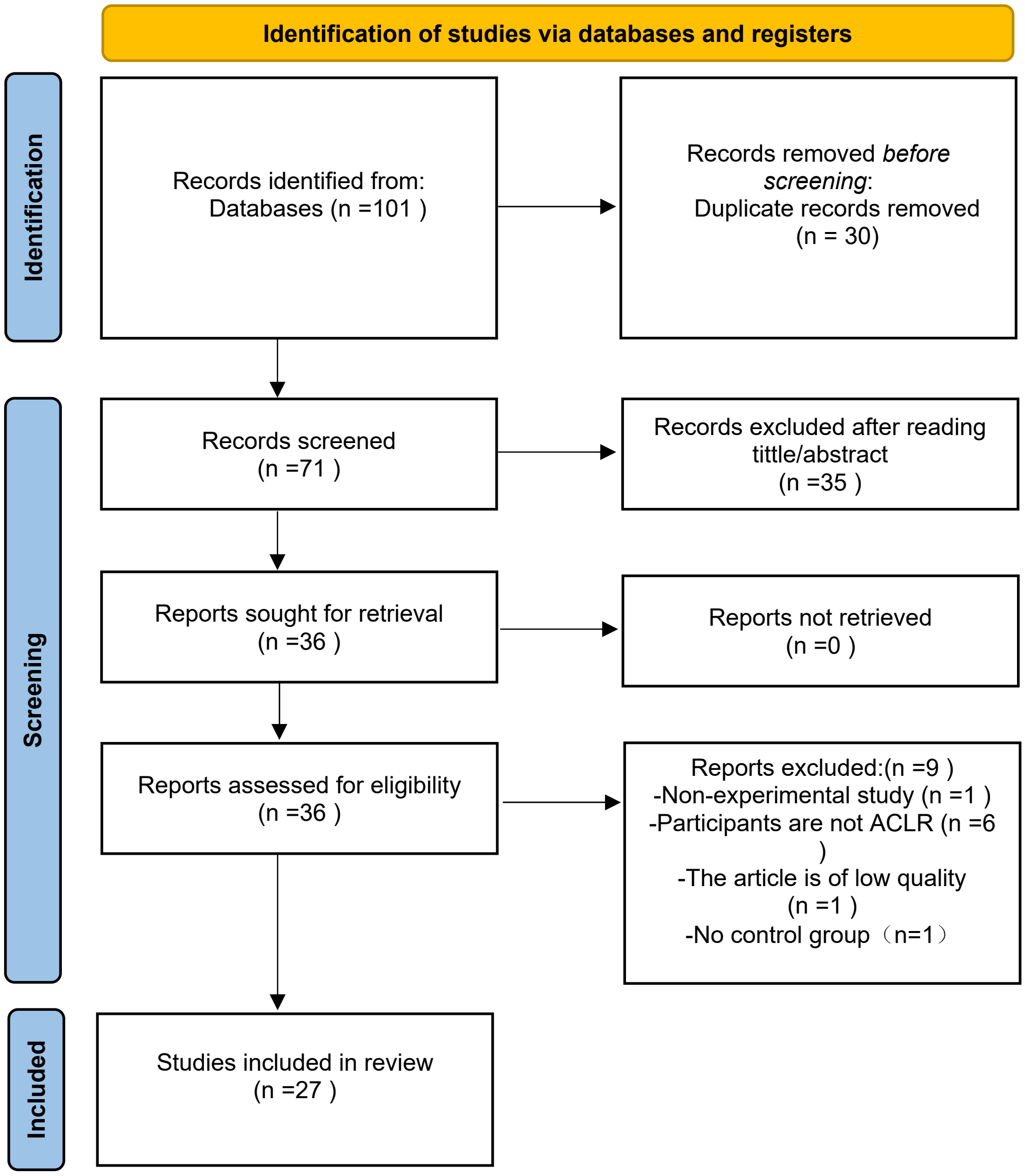

*Consider, if feasible to do so, reporting the number of records identified from each database or register searched (rather than the total number across all databases/registers).

**If automation tools were used, indicate how many records were excluded by a human and how many were excluded by automation tools.

From: Page MJ, McKenzie JE, Bossuyt PM, Boutron I, Hoffmann TC, Mulrow CD, et al. The PRISMA 2020 statement: an updated guideline for reporting systematic reviews. BMJ 2021;372:n71. doi: 10.1136/bmj.n71

For more information, visit: http://www.prisma-statement.org/
