# Supplementary material for: Advances in Research on Brain Structure and Activation Characteristics in Patients with Anterior Cruciate Ligament Reconstruction: A Systematic Review
Source: Brain Sci. 2025 Aug 1;15(8):831. doi: 10.3390/brainsci15080831 (PMC12384111; doi:10.3390/brainsci15080831)
Supplement: Supplementary file 1 [file brainsci-15-00831-s001.zip › Supplementary Table S1.pdf]

PRISMA 2020 for Abstracts Checklist

Section and Topic	Item #	Checklist item	Reported (Yes/No)
**TITLE**			
Title	1	Advances in Research on Brain Structure and Activation Characteristics in Patients after Anterior Cruciate Ligament Reconstruction: A Systematic Review	Yes
**BACKGROUND**			
Objectives	2	This review summarizes the characteristics of brain reorganization in ACLR patients and its association with functional outcomes, aiming to provide a basis for rehabilitation interventions.	Yes
**METHODS**			
Eligibility criteria	3	the included studies: (1) Study details, including author names and publication year; (2) Participant characteristics, including age, gender, injury/postoperative duration, and type of injury/surgery; (3) Technical specifications; (4) Test tasks; (5) Main research findings.	Yes
Information sources	4	Following the PRISMA guidelines, we systematically searched PubMed, Embase, Web of Science, Scopus, Cochrane CENTRAL (2018-2025).	Yes
Risk of bias	5	The ROBINS-I tool was used to assess the risk of bias in 27 observational studies, covering 7 domains including confounding bias, selection bias, etc.	Yes
Synthesis of results	6	Studies were screened using the PICO framework. Inclusion criteria encompassed studies involving ACLR patients, neuroimaging assessments, and controlled designs.	Yes
**RESULTS**			
Included studies	7	Twenty-seven studies were included in the analysis.	Yes
Synthesis of results	8	Brain Activity: Altered activation in sensory cortices, enhanced activation in visual cortices, and reduced efficiency in motor cortices; Enhanced connectivity in the fronto-parieto-occipital regions alongside reduced cognitive-motor neural efficiency.<br>Brain Structure: Reduced volume of the corticospinal tract ipsilateral to the injured limb and decreased thickness in the sensorimotor cortex.<br>Functional Associations: Enhanced activation in sensory integration areas/visual-cognitive regions was associated with improved lower limb functional outcomes.	Yes
**DISCUSSION**			
Limitations of evidence	9	ACLR patients exhibit specific patterns of neuroplastic changes that impact functional recovery. Current research is limited by predominantly cross-sectional designs and methodological heterogeneity. Future studies should integrate neuromodulation techniques to optimize rehabilitation protocols.	Yes
Interpretation	10	ACLR patients exhibit distinct patterns of neuroplastic reorganization involving sensory, motor, visual, and integrative brain networks. These central nervous system alterations significantly influence functional recovery trajectories.	Yes
**OTHER**			
Funding	11	This work was supported by the Beijing Municipal Great Wall Scholar Program (Grant No:	Yes

PRISMA 2020 for Abstracts Checklist

Section and Topic	Item #	Checklist item	Reported (Yes/No)
		CIT&TCD20180335)	
Registration	12	The protocol for this systematic review was prospectively registered in the International Prospective Register of Systematic Reviews (registration number: CRD420251109648).	Yes

From: Page MJ, McKenzie JE, Bossuyt PM, Boutron I, Hoffmann TC, Mulrow CD, et al. The PRISMA 2020 statement: an updated guideline for reporting systematic reviews. BMJ 2021;372:n71. doi: 10.1136/bmj.n71. This work is licensed under CC BY 4.0. To view a copy of this license, visit https://creativecommons.org/licenses/by/4.0/
